# Successful Salvage and Long-Term Survival after Recurrent Malignant Rhabdoid Tumor

**DOI:** 10.1155/2007/53549

**Published:** 2007-07-11

**Authors:** Ryan Horazdovsky, J. Carlos Manivel, Edward Y. Cheng

**Affiliations:** ^1^Department of Orthopaedic Surgery, Medical School, University of Minnesota, Minneapolis, MN 55454, USA; ^2^Department of Laboratory Medicine and Pathology, Medical School, University of Minnesota, Minneapolis, MN 55454, USA

## Abstract

*Purpose*. The objective of this study is to report a case of a rare, highly lethal tumor, extrarenal malignant rhabdoid tumor (EMRT) in a 43-year-old man who initially presented with a local recurrence and is now continuously disease free 14 years after aggressive surgical treatment. The case and literature are discussed.

## 1. INTRODUCTION

Malignant rhabdoid tumor (MRT) was first described as a rhabdomyosarcomatous subtype of Wilm's tumor [1]. Malignant rhabdoid tumors were recognized as a distinct entity in 1981 with extrarenal variants now reported in the CNS, liver, female genital tract, and soft tissues. Extrarenal malignant rhabdoid tumor is a highly lethal, rare tumor with a poor prognosis. A recent report of extracranial, extrarenal rhabdoid tumors estimated a three-year survival of 9% (standard deviation 6%) and an incidence of 0.15 per million children under the age of 15 [2]. The incidence of MRT in adults is likely lower as cases in adults are less frequently reported. The medical literature is replete with papers describing the highly lethal nature of MRT; however, there are reports of longer survivals that range from 72 to 192 months [3–8]. We describe a man with a locally recurrent inguinal malignant rhabdoid tumor diagnosed at age 43 who is continuously disease-free after 14 years. The combination of this patient's age at diagnosis and long survival time following a local recurrence make this case unique.

## 2. CASE REPORT

**Figure 1:**
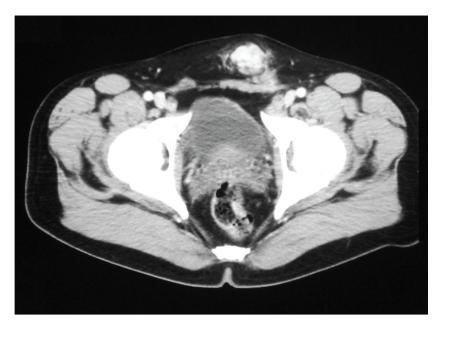
Round region of heterogeneously increased density in anterior abdominal wall just superior to symphysis pubis. This region measures approximately 3 cm in diameter. Postoperative inflammatory changes are noted just inferior to this region.

A 43-year-old man presented with left groin pain, and four months later underwent left herniorrhaphy for presumed inguinal hernia during which a 3.5 cm mass was found. The mass was resected during the herniorrhaphy and diagnosed as an extrarenal malignant rhabdoid tumor. After discussion at our institution's tumor board, the patient was scheduled to undergo 6 rounds of neoadjuvant chemotherapy which included ifosfamide (1.8 g/m^2^ × 5 days), VP-16 (100 mg/m^2^ × 5 days), and G-CSF (390 *μ*g SQ daily × 20 days) followed by surgical excision. After two rounds of chemotherapy and three months after the initial resection, a 3 cm firm, fixed, subcutaneous mass was palpated on the medial aspect of the inguinal incision. CT confirmed the presence of a locally recurrent mass not observed on the previous CT exam performed immediately postresection (Figure 1).

### 2.1. Local excision

The patient then underwent a wide local excision of the 3.0 cm mass, including the abdominal wall, inguinal canal, and testicle, combined with lymph node dissection. Smaller, palpable nodules within the subcutaneous tissue were present. As he had previously had a herniorrhaphy, an aggressive resection of the entire tumor bed was performed. The inguinal canal, left spermatic cord, testicle, and LLQ abdominal wall were removed en bloc. Marlex mesh was used to reconstruct the defect, and extra iliac lymph node dissection was performed. Upon pathological evaluation, the tumor was markedly infiltrative at the edges although the surgical margins were negative and removed lymph nodes were free of tumor. The tumor extended into the actual substance of the upper end of the spermatic cord, but not into the testis or lower end of the spermatic cord.

### 2.2. Histology

**Figure 2:**
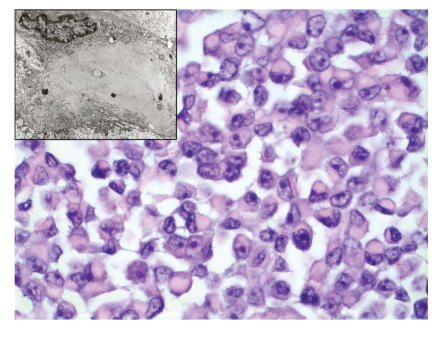
Rhabdoid cells grow in loosely cohesive sheets. The cells are rounded to ovoid, have rounded nuclei with prominent nucleoli; homogeneous, deeply, eosinophilic hyaline cytoplasmic inclusions displace the nucleus laterally (hematoxylin and eosin stain, X 600). A magnified part by electron microscopy shows that paranuclear spherical aggregates of intermediate filaments displace most organelles and the nucleus. No tonofilaments are present (original magnification is X 9660).

The cells were uniform and arranged in solid sheets, infiltrating nests, and cords. The nuclei were oval to round with clear chromatin and a single prominent central nucleolus. The cytoplasm was amphophilic to eosinophilic. In many cells, the nucleus was eccentric, displaced by a bright eosinophilic cytoplasmic inclusion. The tumor had brisk mitotic activity (Figure 2). There were no areas of necrosis. By immunoperoxidase stains, the cytoplasmic inclusions were strongly reactive for vimentin. The tumor cells were also positive for cytokeratin and epithelial membrane antigen. They were nonreactive for leukocyte common antigen and S100 protein. Mucicarmine stain was also negative.

### 2.3. Electronmicroscopy

Electron photomicrographs demonstrated neoplastic cells characterized by enlarged nuclei, prominent nucleoli, and irregular nuclear membrane. There were large whorls of intermediate filaments forming aggregates adjacent to the nucleus (Figure 2). Ultrastructural elements such as desmosomes, intracellular lumen formation, tonofilaments, thin and thick filaments, neurosecretory granules, and cell processes were not observed.

### 2.4. Radiation therapy

Following the operation, the patient underwent external beam radiation treatment and received 6720 cGy in 56 fractions over 40 days with cone-down technique.

### 2.5. Followup status

As of January, 2007, the patient is continuously disease free fourteen years after treatment.

## 3. DISCUSSION

Rhabdoid tumors are characterized by cells with vesicular nuclei, large nucleoli, and variably prominent eosinophilic hyaline cytoplasmic inclusions. Ultrastructurally, the latter consist of whorls of intermediate filaments. This “rhabdoid phenotype” may be present focally in a large variety of other mesenchymal and epithelial malignancies. Tumors in which specific lines of differentiation can be determined should not be designated as rhabdoid tumors, but should be classified according to the specific line observed, such as squamous carcinoma, malignant melanoma, and synovial sarcoma, with rhabdoid features. The rhabdoid phenotype is usually associated with a worse prognosis; nevertheless, these tumors should be classified and treated according to the specific line of differentiation observed. Such tumors predominate in adults. Therefore, the diagnosis of malignant rhabdoid tumor is a diagnosis of exclusion, after other possibilities have been excluded through adequate sampling, ultrastructural, and immunohistochemical studies. After such tumors with “rhabdoid phenotype” have been excluded, there is a group of malignant rhabdoid tumors in the soft tissues that predominates in infants and children; however, the overall range is wide and they can also occur in adults.

Histologic diagnosis of extrarenal MRT is aided by a variety of immunostains. Indeed, as observed in our case, these tumors display polyphenotypic immunohistochemical profiles. A variety of antigens may be detected in the cells of “pure” extrarenal MRT, including epithelial, mesenchymal, and neural antigens. The rhabdoid cells are characterized by aggregates of intermediate filaments comprised of vimentin and cytokeratin [9]. A study of eighteen soft tissue MRTs showed that 94% were positive for vimentin and 59% for pancytokeratin [10]. The keratin profile is more restricted than that of epithelioid sarcoma as no tonofilaments are identified in MRT by electron microscopy. The immunoprofile of extrarenal MRT overlaps that of the so-called “proximal epithelioid sarcoma.” Existence of the latter as a clinicopathologic entity has been questioned; some authors believe that it represents a variant of extrarenal MRT. Interestingly, some examples of classic epithelioid sarcoma display aberrations of 22q similar to those described for extrarenal MRT. The large cell size and marked cytologic atypia associated with the so-called “proximal epithelioid sarcoma” were not seen in our case.

Loss of immunoreactivity for INI1 antibody is valuable in confirming the diagnosis of renal or extrarenal MRT versus other tumors with focal rhabdoid appearance [11]. INI1 is part of an ATP-dependent chromatin remodeling complex expressed in all tissues [12, 13]. INI1 is a product of the hSNF5/INI1 tumor suppressor gene that is frequently mutated or deleted in MRTs. Cytogenetic study of malignant rhabdoid tumors (renal and extrarenal) reveals a region of common deletion at 22q11, reputedly the locus of hSNF5/INI1. Analysis of chromosome 22q has recently been used as an aid to the diagnosis of rhabdoid tumors [14]. In MRT cell lines, reexpression of the hSNF5 gene induces G1 cell cycle arrest and activation of senescence-associated proteins [15–18].

In a recent study, 6 of 38 cases of MRT retained immunohistochemical expression of INI1 and failed to show any genetic alteration at the hSNF5/INI1 locus. After excluding other diagnoses, their morphology and combined mesenchymal and epithelial patterns were strongly indicative of the genuine rhabdoid nature of these tumors. Therefore, mutations involving other members of the chromatin remodeling complex may result in functional consequences similar to hSNF5/INI1 loss of function [19]. Indeed, Fruhwald et al. reported recently a family with MRT affecting 2 siblings without hSNF5/INI1 germline mutations, suggesting the existence of a second predisposing locus for MRT [20]. Unfortunately, no cytogenetic study was performed in our case and no cryopreserved tissue is available for molecular analysis.

Common treatment protocols have been attempted for rhabdoid tumors and Wilms' tumor. The British UKW2 and UKW3 WILMS' tumor treatment protocols were employed in 21 patients with renal rhabdoid tumors reported to the National Registry of Childhood Tumors (NRCT) between 1987 and 1999. The chemotherapy regimen recommended consisted of vincristine 1.5 mg/m^2^ weekly × 11, then every 3 weeks, together with actinomycin D 1.5 mg/m^2^ and doxorubicin 30 mg/mg/m^2^ given at 3-week intervals for a total of 1 year. Patients with abdominal stage III tumors were to receive 30 Gy radiotherapy to the flank. Median age at diagnosis in this group was 1.7 years with a 5-year survival of 35% [2].

Other chemotherapy regimes for MRT have included combinations of cisplatinum, cyclophosphamide, adriamycin, and VP-16 [21–23]. The German Society of Pediatric Oncology Protocol consisted of HIT, procarbazine, ifosfamide, VP-16, methotrexate, cytosine-arabinoside, and cisplatin [21]. These protocols were reported after our patient was treated. Unfortunately, most of them are based on anecdotal reports. Limited clinical trial data exists for extrarenal MRT. Historically, extrarenal MRT has been shown to be highly lethal with survival near 9% at 3 years [2]. We attempted neoadjuvant chemotherapy in our patient with agents known to have efficacy in sarcomas. Despite this, his disease relapsed while on treatment. An aggressive surgical excision was then performed along with radiation therapy in an attempt to eradicate his localized disease. Our experience demonstrates that despite the extremely poor prognosis associated with extrarenal MRT, an aggressive surgical excision and radiation therapy in the setting of localized disease can result in long-term survival.
